# Influence of Lamina Types and Combinations of Deep Marine Shale on Reservoir Quality in Zigong Block of Southern Sichuan Basin

**DOI:** 10.1021/acsomega.4c06917

**Published:** 2024-11-04

**Authors:** Xiangyang Pei, Xizhe Li, Wei Guo, Haoyong Huang, Yize Huang, Qimin Guo, Mengfei Zhou, Longyi Wang, Sijie He, Wenxuan Yu

**Affiliations:** † Research Institute of Petroleum Exploration & Development, PetroChina, Beijing 100083, China; ‡ National Energy Shale Gas R&D Experimental Center, Beijing 100083, China; § Institute of Porous Flow & Fluid Mechanics, University of Chinese Academy of Sciences, Langfang, Hebei 065007, China; ∥ Exploration and Development Research Institute, PetroChina Southwest Oil & Gas Field Company, Chengdu 610051, China; ⊥ Department of Energy and Mineral Engineering, EMS Energy Institute, and G3 Center, The Pennsylvania State University, University Park, Pennsylvania 16802, United States; # Chongqing University of Science and Technology, Chongqing 401331, China

## Abstract

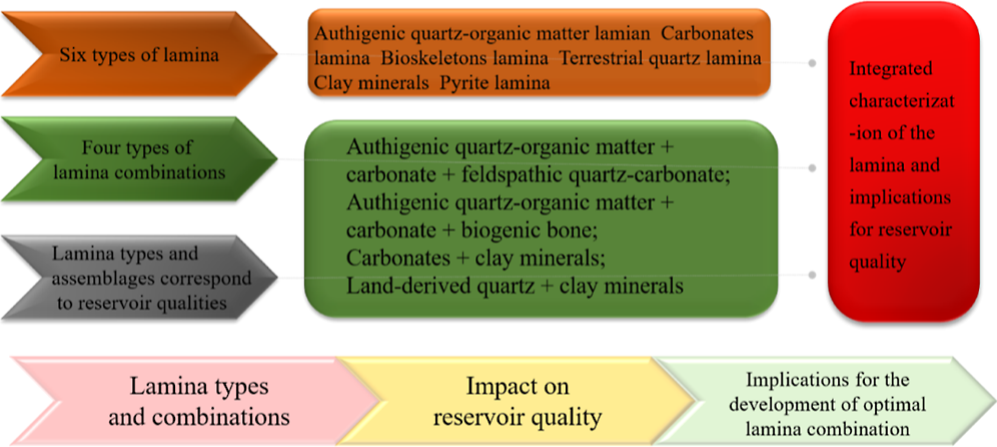

The influence of the type of lamina and its combination on the reservoir quality in deep-sea shales of the Zigong Block in the southern Sichuan Basin was investigated. The shale reservoirs in the Wufeng and Longmaxi Formations were characterized in detail by employing a variety of advanced techniques such as full-scale thin section imaging, micro-X-ray fluorescence spectroscopy (Micro-XRF), field emission scanning electron microscopy, and a quantitative mineral evaluation system (QEMSCAN). This study systematically analyzed the structural characteristics of different types of lamina and their effects on the reservoir porosity, permeability, and gas content. The findings reveal that the type and combination of laminae significantly affect the reservoir quality of shale. Among the identified combinations, the “authigenic quartz–organic matter + carbonate + felsic–carbonate” lamina combination demonstrates the most favorable characteristics, with higher organic content, brittle mineral content, and porosity, making it the optimal “sweet spot” for shale gas development. This research provides crucial theoretical insights into the formation mechanisms of deep marine shale gas reservoirs and supports further advancements in shale gas exploration and development.

## 1 Introduction

As the largest commercial area of shale gas in China,^1^ the Sichuan Basin has already witnessed large-scale production of medium-shallow marine shale gas in several blocks such as Changning, Weiyuan, Zhaotong, and Fuling.^2^ With the continuous proceeding of exploration and development, deep marine shale gas will become the main force for the increasing of shale gas reserves and production in China.^3,4^ Due to good quality and continuous and stable distribution at the depth ranging from 3500 to 4500 m, the Ordovician Wufeng Formation–Silurian Longmaxi Formation shale reservoirs in the Zigong Block of southern Sichuan Basin are currently the main areas for deep shale gas exploration and development in China.^5,6^ The exploration and development of deep shale gas is still in the stage of exploration, with the researches mainly focusing on potential evaluation,^7,8^ exploration, and development prospect prediction and development engineering technological tackling, and there is still a lack of systematic understanding on the characteristics and formation mechanisms of good-quality reservoirs.^9,10^ The black shale of the Paleozoic Wufeng Formation–Longmaxi Formation is widely distributed in the southern Sichuan area, and the lamina is widely developed in the shale. In addition, the type and combination of the lamina not only reflect the dynamic changes of the depositional environment but also directly affect the key attributes of the reservoir, such as the pore structure, permeability, and material composition. In shale gas reservoirs, the distribution and development degree of the lamina are usually closely related to the organic matter enrichment and preservation conditions, which directly affect the reservoir quality. The study of shale lamina type and combination characteristics can help predict the reservoir physical properties and resource potential, so an in-depth investigation of the shale lamina type, genesis, and mechanism of the impact on reservoir quality is of great significance in enhancing the benefits of the development of shale oil and gas reservoirs.

Lamina is the basic unit in a sedimentary layer, which refers to the smallest or thinnest primitive sedimentary layer in sedimentary rock that can be distinguished with naked eyes, and its thickness is usually less than 1 cm.^10^ As a unique structure in shale layers, lamina corresponds to different diagenetic processes and responses in the burial process, and its types, structures, and combinations are under the control of sedimentation, so that it impacts the reservoir property, gas-bearing property, and friability of the shale reservoir.^11−13^ At present, there are some differences between domestic and foreign lamina researches. Foreign scholars pay attention to the morphology, continuity, and structural attributes of lamina.^14,15^ For example, Schieber explored the formation mechanism of lamina by means of flume simulation experiments^14^ and proved that under the action of flocculation, fine materials such as clay minerals can be transported through the bottom bed, thus forming low-angle cross bedding. Chinese scholars focus on the types, developmental characteristics, formation mechanisms, heterogeneity, interfacial contact relationships, and combinations of lamina. They made use of experimental data to trace the sedimentary environment for the formation of lamina and then established the relationship model between lacustrine shale lamina and shale oil and gas enrichment.^15^ At present, however, the lamina in marine shale is less researched, the classification scheme is not unified, and less attention is paid to the influence of the lamina structure on reservoir quality. Therefore, the current research shall be strengthened in the following three aspects, i.e., the lamina types and combinations developed in deep shale, the influence of different types of laminae and their combinations on the reservoir properties and permeability of shale, and the distribution intervals of the optimal lamina combination.

In the Wufeng Formation–Longmaxi Formation of the Zigong Block, grayish black and dark gray shale are widely developed, and the rock particles are so small that it is difficult to directly identify the growth characteristics of shale lamina in the core scale. To this end, this paper applied a variety of high-precision characterization techniques, including full-scale rock thin section imaging, micro-X-ray fluorescence spectrum (Micro-XRF) analysis, field emission scanning electron microscopy (FE-SEM), and a scanning electron microscopy-based quantitative mineral evaluation system (QEMSCAN) to dissect the structural characteristics of lamina, thus realizing the fine classification of lamina type and combination. Based on this, the controlling effects of different lamina combinations on reservoir quality were discussed, and the optimal lamina combination in shale reservoirs and its distribution were determined. It aims to provide a solid theoretical basis for further understanding shale gas enrichment mechanisms in the future.

## 2 Geological Overview

The southern Sichuan area is located in the southwestern margin of Upper Yangtze Platform in the southern China, and it is bounded with the Daliang Mountains on the east, the Silurian denudation line of Leshan–Longnvsi paleo-uplift on the south, and the Huaying Mountains on the west, covering an area of about 4 × 10^4^ km^2^. Well Fuye 2 lies at the Gaoshikan syncline of the southwestern Sichuan low fold belt in Doushan Town, Fushun County, Sichuan Province, and takes the Wufeng Formation–Longmaxi Formation as the target layer. During the Late Ordovician to the Early Silurian, the global sea level rose rapidly, the ancient land uplifted in a large scale, and consequently, the southern Sichuan area was overall in a deep-water shelf environment surrounded by the central Sichuan uplift, the central Guizhou uplift, the Xuefeng upper uplift in the South China, and the local underwater high lands, where a set of organic-rich thick black shale was deposited and evolved into a shallow shelf and tidal flat coastal environment successively to the direction of the paleo-land and paleo-uplift.^4^ The Zigong Block is located in the low steep fold belt of southern Sichuan Basin, where the Wufeng Formation—the first submember of the first Member of Longmaxi Formation (hereinafter referred to as Long 1_1_ submember)—is the target layer of shale gas exploration and the lithology is grayish black/dark gray siliceous, calcareous, and silty shale.^9^

## 3 Experimental Sample and Method

### 3.1 Sample Preparation

The samples were prepared from the cores taken from Well Fuye 2 in the Zigong Block. The sampling horizon was the Wufeng Formation–Long 1_1_ submember to the Long 1_4_ submember. According to the experimental purposes, four types of samples were prepared. A 50 mm × 70 mm × 0.03 mm core thin section was prepared by cutting it perpendicular to the bedding plane for thin section analysis and XRF in situ element scanning. A blocky rock sample with a diameter less than or equal to 25 mm and thickness less than 10 mm was prepared by cutting it perpendicular to the bedding plane and then polished with argon ion and coated with carbon for mineral scanning and analysis by means of FE-SEM and QEMSCAN. A plunger sample was acquired for pore and permeability measurement. A powder sample was prepared from the residual core to determine organic abundance by measuring TOC and identify mineral types and contents and principal elements in the rock accurately by means of X-ray diffraction.

### 3.2 Experimental Method

First, full-scale core thin section imaging was performed to understand the overall structural characteristics. Then, a polarized light microscope was used to observe the microscopic texture and mineral distribution in the core, and QEMSCAN and Micro-XRF mineral analyses were carried out to identify the mineral compositions and chemical components of the lithology more accurately. Finally, FE-SEM was applied to observe the characteristics of the reservoir space, and the pulse decay method (PDP) and the liquid saturation method were used for the permeability and porosity measurement. In this way, the texture, structure, mineral composition and distribution, elemental distribution, and reservoir space characteristics of lamina were revealed step by step from a macroscopic to a microscopic scale, which provides important data and basis for reservoir evaluation.

The QEMSCAN combines dual-probe X-ray energy spectrum with backscatter high-resolution imaging, so it can determine the type, content, size distribution, and contact relationship of minerals by directly measuring rock samples. In this study, a Quanta FEG 450 FE-SEM system made by the FEI Company was used, and it can generate high-resolution backscatter electronic images. Also, by virtue of the QEMSCAN analysis system equipped in this instrument, the mineral compositions in different lamina and their distribution were analyzed successfully. First, the rock samples were argon ion polished and carbon plated and then placed under a scanning electron microscope. Specific areas were selected for image scanning and stitching to obtain a large-area scanning image (MAPS). Then, the elements were analyzed using the X-ray energy spectrum analysis tool, and the mineral information was compared to obtain a sample test diagram with mineral compositions. Finally, the scanning electron microscope was adjusted to a voltage of 5 kV, a current of 0.4 nA, and a working distance of 4 mm for high-resolution backscattered electron two-dimensional aperture imaging. Micro-XRF is an in situ rapid evaluation technique that makes use of the energy-dispersive analysis method to accurately characterize rock compositions and textures. This study adopted the M4 TORNADO Micro-XRF tool made by the Bruker Company of Germany, which excites the sample with micrometer-sized light spot of X-ray, which is generated by focusing multiconductivity capillary on the target material, so as to realize the element imaging of rock compositions. This technology can be used for accurate analysis of rock compositions and textures. The whole scanning process is under precise control of a computer, and the three-dimensional scanning is completed at high precision and small step displacement to ensure the accuracy and reliability of the analysis results.

## 4 Lamina Types and Combinations

### 4.1 Basic Lamina Types

**Figure 1:**
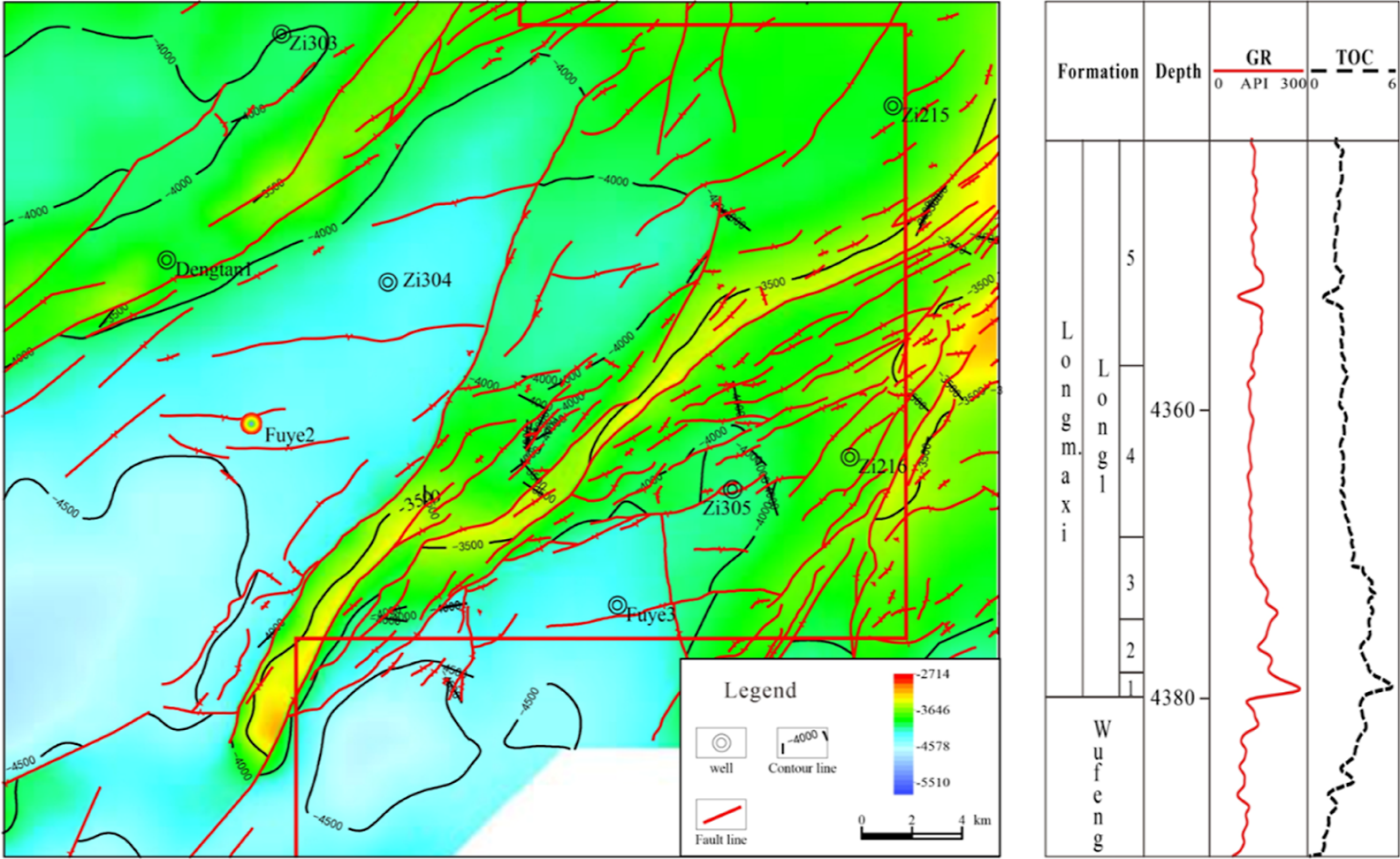
Structural and stratigraphic characteristics in the study area.

According to particle size, shale lamina can be classified into clayey lamina (particle size smaller than 31.2 μm) and silty lamina (particle size ranging from 31.26 to 62.5 μm).^6^ Clayey lamina is mainly composed of authigenic quartz and organic matter with a little carbonate mineral, and silty lamina is rich in carbonate mineral, terrigenous clastic quartz, clay mineral, and pyrite. In view of the differences in mineral compositions, clayey lamina can be further classified into three types, i.e., authigenic quartz–organic lamina, siliceous lamina, and clay mineral lamina, and silty lamina can be further classified into four types, i.e., felsic–carbonate lamina, carbonate lamina, terrigenous clastic quartz lamina, and pyrite lamina (Figure 1).

#### 4.1.1 Authigenic Quartz–Organic Lamina

**Figure 2:**
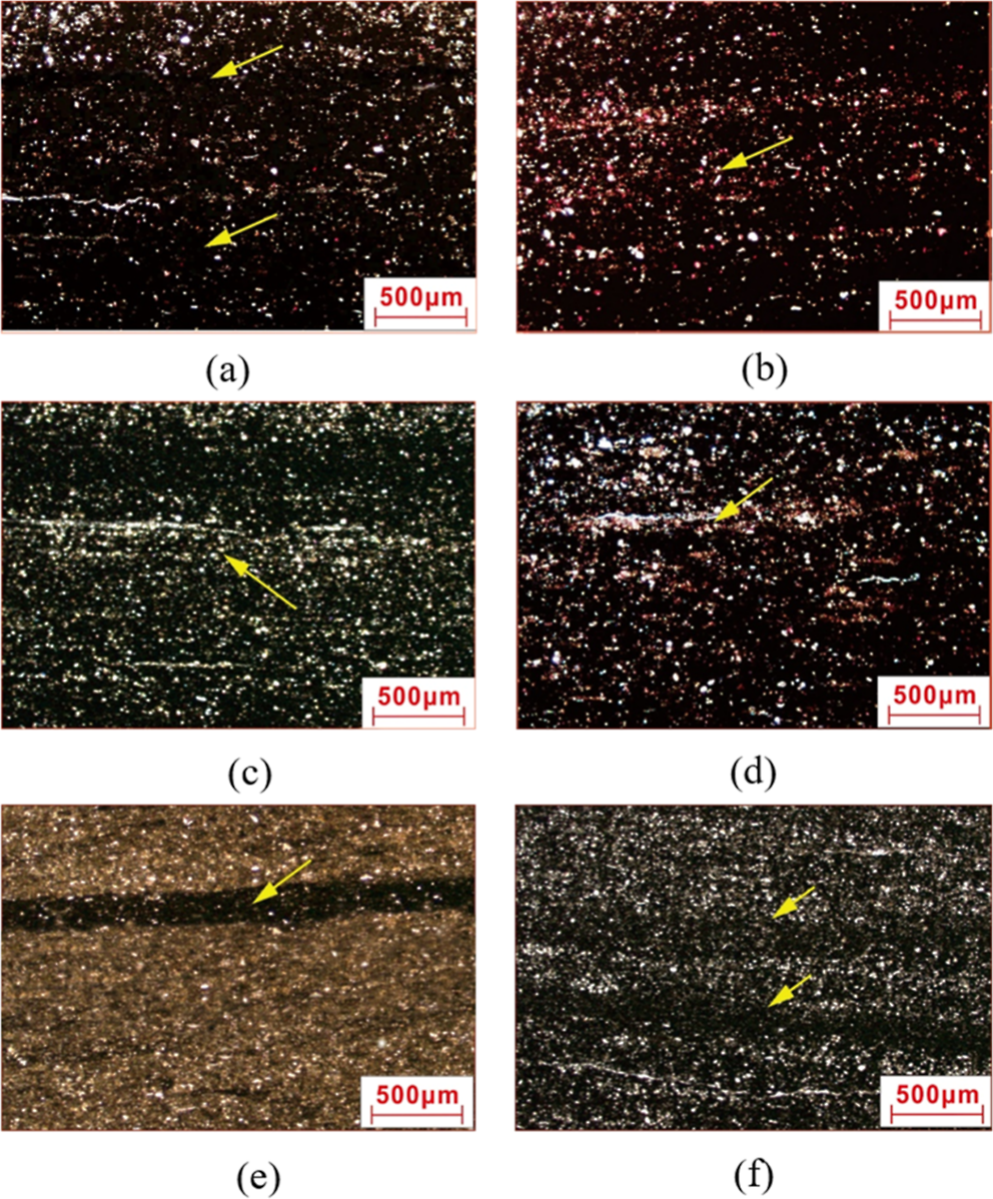
Rock thin sections of Long 1_1_ typical lamina in the Zigong Block. (a) Authigenic quartz–organic lamina; (b) felsic–carbonate lamina; (c) terrigenous clastic lamina; (d) clay mineral lamina; (e) pyrite lamina, with calcareous bioclastic in the lower section; and (f) terrigenous quartz with interbedded thin double-clay mineral rhythmic lamina.

**Figure 3:**
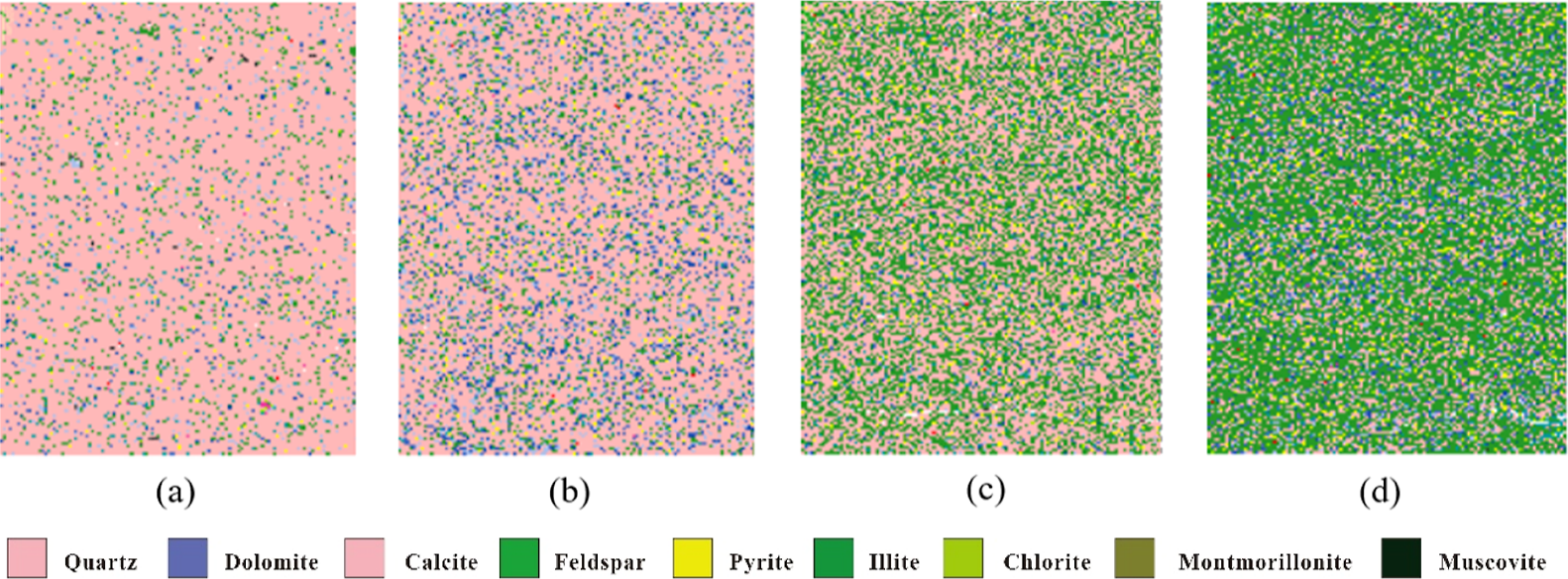
Mineral distribution characteristics in different types of lamina by QEMSCAN. (a) Its quartz content exceeds 83.2% and calcite and dolomite content is about 5.6%. (b) Its felsic mineral content is about 76% and carbonate mineral content is about 27%. (c) Its quartz and illite contents are high up to 57.3% and 23%, respectively, and the mica content increases to 3%, indicating an increase in the terrigenous input. (d) Its illite content is about 26.7%, quartz content is about 36.6%, calcite and dolomite content is about 15.4%, and mica content is about 4%.

Under a plane light microscope, authigenic quartz–organic lamina presents blackish brown and continuously distributed horizontal layered textures enriched with organic matters. Its thickness is mainly in the range of 1.3–20 mm (Figure 2a). The authigenic micritic quartz particles in this type of lamina are diverse in morphology and tiny in size, about 1–2 μm. Organic matter is dispersive to form a connected network structure. The QEMSCAN mineral analysis results show that its quartz content exceeds 83.2% and its calcite and dolomite content is about 5.6% (Figure 3a).

#### 4.1.2 Felsic–Carbonate Lamina

Under a plane light microscope, felsic–carbonate lamina presents as grayish white. It is about 4–7 mm wide (Figure 2b). Rock particles are large sized with poor sorting and angular to subangular shape, and they are mainly in the silty level. It is mainly composed of felsic and carbonate minerals, which are related to volcanic materials. Some of carbonate compositions are replaced with quartz, containing a small number of siliceous spots, which indicates the growth process of the siliceous mineral after the devitrification of volcanic ash. The QEMSCAN mineral analysis results show that its felsic mineral content is about 76% and its carbonate mineral content is about 27% (Figure 3b).

#### 4.1.3 Terrigenous Clastic Lamina

Under a plane light microscope, terrigenous clastic lamina presents as a light color. Its thickness ranges from 0.7 to 4.0 mm (Figure 2c). Its texture is dominated by silty clastic, mainly including terrigenous angular quartz siltstone and mica fragments, and its particle size is mainly in the range of 26–50 μm. There are abundant clay minerals in terrigenous clastic lamina, which are widely distributed as interstitial materials. Organic matter is mostly dispersed in the banded or lumpy form between siltstone particles, and most of them are unconnected. This type of lamina changes gradually with the siltstone content and brightness decreasing upward. The QEMSCAN mineral analysis results show that its quartz and illite contents are high up to 57.3% and 23%, respectively, and its mica content increases to 3%, indicating an increase in the terrigenous input (Figure 3c).

#### 4.1.4 Clay Mineral Lamina

Under a plane light microscope, clay mineral lamina presents as blackish brown. It is as thin as only 0.5 mm. This type of clay mineral lamina has the rhythmic characteristics of double-clay mineral lamina (Figure 2d). It is composed of black clay mineral and organic pellet, with a little organic matter, terrigenous quartz, and mica fragment. The long axis of the organic pellet is parallel to the lamina. The QEMSCAN mineral analysis results show that its illite content is about 26.7%, quartz content is about 36.6%, calcite and dolomite content is about 15.4%, and mica content is about 4% (Figure 3d).

#### 4.1.5 Pyrite Lamina

Under a plane light microscope, pyrite lamina presents as black. It is relatively flat and straight, but its thickness varies in the range of 0.2–0.5 mm (Figure 2e,f). Coarse nodules are developed locally in the shape of a patch with the growth surface of an automorphic crystal. On the surface of the patch develops quartz and calcite, and the crystal is platy prismatic in the direction perpendicular to the crystal surface, which is the result of the metasomatism during diagenesis.

### 4.2 Lamina Combinations and Their Distribution

The sources rocks formed in different sedimentary environments are different in lamina density and lamina combination type.^16^

#### 4.2.1 Authigenic Quartz–Organic Matter + Carbonate + Felsic–Carbonate

**Figure 4:**
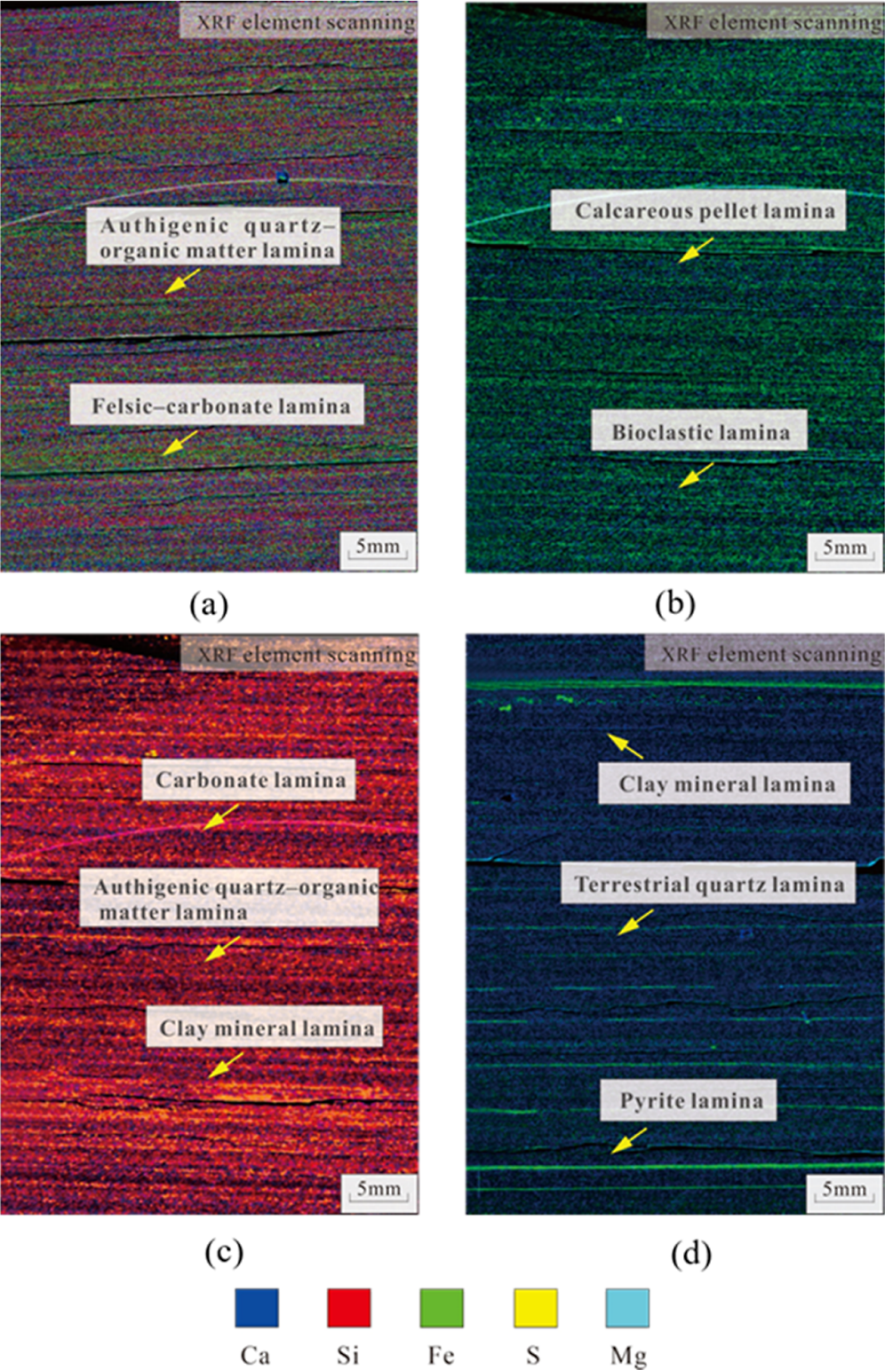
Lamina combinations in the Long 1_1_ submember of Zigong Block and their XRF element scanning characteristics. (a) Authigenic quartz–organic lamina; (b) felsic–carbonate lamina; (c) terrigenous clastic and clay mineral lamina; and (d) pyrite lamina.

This type of shale is mainly developed in the first sublayer of the Long 1_1_ submember. It is grayish black and rich in organic matter with an average TOC of 5.15%. The authigenic quartz–organic lamina is thicker and the carbonate lamina is thinner, both of which alternate with each other frequently in a regular pattern with a flat and straight interface. Thick and wide felsic–carbonate mixed lamina is developed locally (Figure 4a). XRF element scanning analysis results show that carbonate lamina is dominated by element Ca, authigenic quartz–organic lamina is dominated by element Si, and felsic–carbonate lamina contains Al, Si, Ca, and Fe (Figure 4a).

#### 4.2.2 Authigenic Quartz–Organic Matter + Carbonate + Bioclastic

This type of shale is mainly developed in the second sublayer of the Long 1_1_ submember, and it is grayish black. Its TOC content averages 4.46%. Authigenic quartz–organic lamina is developed with interbedded thin carbonate lamina. Bioclastic and calcareous pellet laminae are developed locally (Figure 4b). XRF element scanning results show that bioclastic lamina is dominated by element Si, while calcareous pellet lamina is dominated by element Ca, followed by Si (Figure 4b).

#### 4.2.3 Carbonate + Clay Mineral

This type of shale is mainly developed in the third sublayer of the Long 1_1_ submember, and it is grayish black. Its TOC content averages 3.9%. On the whole, no lamina structure is observed. Carbonate mineral and quartz are scattered, and only thin carbonate lamina and clay mineral lamina are developed. In this type of lamina combination, the content of silty quartz begins to increase and the particle size increases, indicating an increase in the content of terrigenous clastic. XRF element scanning analysis results show that the contents of Ca and Fe in this type of shale are 54% and 13%, respectively.

#### 4.2.4 Terrigenous Clastic + Clay Mineral

This type of lamina is commonly developed in the fourth sublayer of the Long 1_1_ submember. It is dark gray and has a low organic matter content with an average TOC of 2.6%. The particle size increases, and the content of terrigenous silty quartz increases greatly. The terrigenous silty quartz lamina is with interbedded thin clay lamina. In the shale with this type of lamina combination, the mineral is dominated by terrigenous quartz, and the clay mineral content is high. XRF element scanning results show a Ca content of 69% and a Mg content of about 10%.

## 5 Influence of Different Types of Laminae on Reservoir Quality

### 5.1 Difference in Pore Types

**Figure 5:**
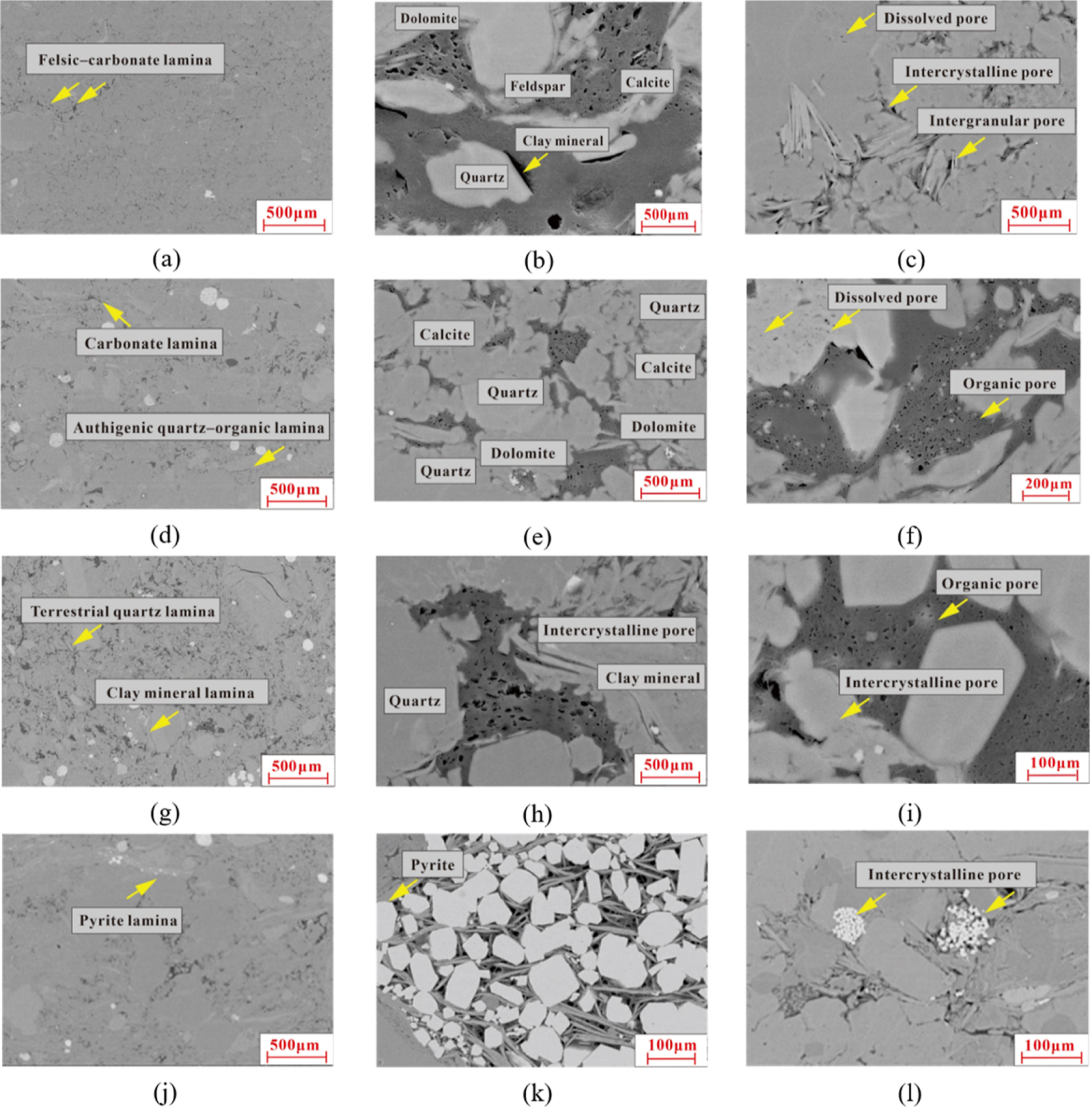
Microscopic pore characteristics in Long 1_1_ authigenic quartz–organic lamina, felsic–carbonate lamina, terrigenous clastic lamina, clay mineral lamina, and pyrite lamina in Zigong Block. (a) Felsic–carbonate lamina. (b) Intercrystalline, intergranular, and dissolved pores developed in felsic–carbonate lamina. (c) In felsic–carbonate lamina, quartz, feldspar, calcite, and dolomite are distributed alternatively with clay mineral as intergranular filling. (d) Carbonate lamina and authigenic–organic lamina are filled with micritic quartz. (e) In felsic–carbonate lamina, carbonate mineral alternates with micritic quartz. (f) In carbonate lamina, mineral dissolved pores and organic pores are developed. (g) Terrigenous quartz lamina and clay mineral lamina. (h) In terrigenous clastic lamina, intergranular pores are developed in clastic quartz and clay mineral. (i) In clay mineral lamina, intercrystalline pores and organic pores are developed. (j) Pyrite lamina. (k) Pyrite lamina is dominated by the automorphic crystal, filling clay mineral particle pores. (l) Intercrystalline pores are developed in the crystal.

The pores are mainly concentrated in the vicinity of the adsorbed components, while the pore space is dynamically evolving, with a normal evolution of the cis-layer in terms of pore direction and shape.^17−19^ In felsic–carbonate lamina, inorganic pores are mainly developed, including intergranular pores, intercrystalline pores, and dissolved pores, while organic pores are developed locally. Mineral particles are distributed in a chaotic pattern with poor sorting, and the space between them is filled with clay mineral (Figure 5a,b). The inorganic pores in this type of lamina are mainly classified into three categories, i.e., intergranular pore developed between mineral particles, intercrystalline pore developed between clay mineral crystals (Figure 5c), and dissolved pore developed in feldspar, calcite, and scattered dolomite, all of which are interconnected with microfractures to constitute good reservoirs.

In carbonate lamina, dolomite and calcite particles are distributed in a banded form, and the intergranular spaces are filled with micritic quartz (Figure 5d,e), with a little pyrite. In this type of lamina, there are mainly two types of pores. A large number of dissolved pores are developed in dolomite and calcite, and abundant organic pores are developed between micritic quartz particles (Figure 5f). In authigenic quartz–organic lamina, organic pores are developed; abundant diagenetic micritic quartz is distributed in ellipsoidal and sheet forms with organic matters as fillings between them; and honeycombed organic pores are quite developed.

**Figure 6:**
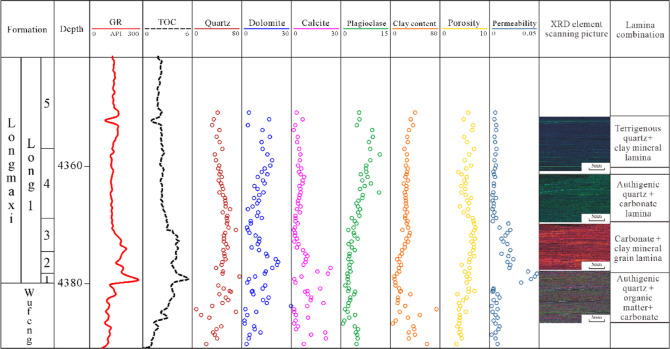
Composite column of Long1_1_ shale in Zigong Block.

In terrigenous quartz lamina and clay mineral lamina, inorganic pores are mainly developed. Terrigenous quartz lamina is rich in terrigenous clastic quartz and clay mineral, and its pores are mainly intergranular pores (Figure 5g,h). In clay mineral lamina, a large number of pores develop between clay mineral crystals (Figure 6j). Pyrite lamina is dominated by automorphic crystals with a little framboidal aggregate content, and a few intergranular pores are developed between crystals (Figure 5k,l). The automorphic pyrite crystals formed during the diagenesis period support the preservation of pores (Figure 5l)

### 5.2 Difference in Reservoir Quality

Different types of lamina combinations control the vertical lithological change of shale, resulting in differences in hydrocarbon generation capacity, reservoir capacity, and fracability of shale reservoir.

The “authigenic quartz–organic matter + carbonate + felsic–carbonate” lamina combination is developed in the first sublayer of the Long 1_1_ submember. In addition, the corresponding shale interval has an average TOC of 5.1%, an average brittle mineral content of 78%, an average porosity of 5.5%, a maximum permeability of 0.046 mD, and an average gas content of 7.4 m^3^/t.

The “authigenic quartz–organic matter + carbonate” lamina combination is developed in the second sublayer of the Long 1_1_ submember. In addition, the corresponding shale interval has an average TOC of 4.3%, average brittle mineral content of 72%, average porosity of 5.8%, maximum permeability of 0.024 mD, and average gas content of 6.1 m^3^/t.

The “carbonate + clay mineral” lamina combination is developed in the third and fourth sublayer of the Long 1_1_ submember. Its TOC content is lower, with an average of 2.8%, average brittle mineral content is 85%, average porosity is 4.6%, maximum permeability is 0.018 mD, and average gas content is 2.4 m^3^/t.

The dark gray massive shale developed in the upper part of Wufeng Formation has an average TOC of 4.3% and a high brittle mineral content, with an average of 77%, an average porosity of 4.3%, and a permeability of 0.008 mD.

## 6 Discussion

### 6.1 Formation Mechanisms of Different Types of Laminae

A large-scale volcanic activity happened in the Upper Yangtze area during the Late Ordovician to the Early Silurian, and volcanic ash got enriched in the sedimentation period of the Wufeng Formation–Longmaxi Formation.^20,21^ In the early sedimentation period of the Rhuddanian (corresponding to the LM1–LM3 graptolite belt), the Yangtze Plate was structurally in a relatively stable condition with a small uplift of the paleo-uplift. After the Hirnantian glaciation, upwelling currents became more active, leading to a flood of nutrients into the Yangtze Basin, which stimulated the recovery of marine organisms on a large scale. In the meantime, a large amount of volcanic materials erupting during the volcanic activity period dropped into the water and released nutrients (e.g., iron salt) to form a rich sea basin, which can cause algal blooms in a short time to facilitate the formation of authigenic quartz–organic lamina.^21^ During strong transportation, silty volcanic materials deposited on land in the early stage were transported to the deep water area to form felsic–carbonate lamina. During weak transportation, clayey sediments were dominant and a large amount of volcanic materials settled in the sea basin and adsorbed clay to form flocculants. Then, the flocculants were transported through the bottom flow to the deep water area, where they were mixed into the fine-grained sediments in the form of intermittent lens.^22^ In the process of diagenesis, altered siliceous lamina could be formed after devitrification of vitric volcanic debris.^23^ In the early Rhuddanian to Aeronian stage (corresponding to the LM5–LM8 graptolite belt), as the Cathaysian Plate collided with the Yangtze Plate continually, the paleo-uplift uplifted again,^24^ the large-scale transgression caused by the melting of the ice sheet ended, and regional regression resulted in sea-level decline.^8^ As the input of terrigenous clastic quartz and clay mineral increased, terrigenous quartz lamina was formed with interbedded clay mineral lamina locally. In this period, oscillating regressions and volcanic activities resulted in great difference in the paleo-productivity of water body in different stages.^8^ In the upper section of the third sublayer of Long 1 Member, the TOC content exceeds 3%, but the input of terrigenous materials results in the scattered distribution of organic matters in shale^25^ (Figure 6).

### 6.2 Genetic Difference in the Reservoir Capacity of Different Types of Laminae

The strong hydrodynamics during sediment transportation leads to large particle size of continental silty volcanic materials and poor sorting, so abundant intergranular pores can be formed between particles in felsic carbonate lamina (Figure 5b). In the process of diagenetic burial, the organic acids released from organic matters through thermal evolution can dissolve the feldspar, calcite, and dolomite in felsic–carbonate lamina to form dissolved pores (Figure 5e,f). In the meantime, bitumen fills felsic carbonate lamina to form organic pores (Figure 5f). In terrigenous quartz lamina, there are intergranular pores between silty quartz particles, but quartz particles are scattered in the matrix to provide poor support for organic pores (Figure 5h), so the organic pores can be hardly preserved. In clay mineral lamina, intercrystalline pores between clay mineral crystals are dominant (Figure 5i).

### 6.3 Optimal Lamina Combination and Distribution

The comprehensive analysis results indicate that from the perspective of reservoir quality, the “authigenic quartz–organic matter + carbonate + felsic + carbonate” lamina combination is the best, followed by the “authigenic quartz–organic matter + carbonate + bioclastic” lamina combination and the “carbonate + clay mineral” lamina combination takes the third place. The quality optimization and classification are mainly based on the following aspects:

(1) High organic content. “Authigenic quartz–organic matter + carbonate + felsic + carbonate” lamina and “authigenic quartz–organic matter + carbonate + bioclastic” lamina have enriched organic matters with TOC content over 4%, so they are excellent source rocks. The “carbonate + clay mineral” lamina has an average TOC of 3.6%, and it is relatively good source rock (Figure 6).
(2) Easily modified mechanical properties. The shale in the laminae mainly developed in the first, second, and third sublayers of the Long 1 Member has an average brittle mineral content of 83.2%, 76%, and 57.3%. As the main brittle mineral in shale reservoirs, quartz is mainly classified into two types, i.e., terrigenous quartz and authigenic quartz.^26−28^ The current research results show that these two types of quartz make different contributions to reservoir fracability. The terrigenous quartz is mainly silty and scattered in the matrix in a floating form, so its contribution to shale fracability is limited (Figure 5h). The authigenic quartz is mostly submicron sized and constitutes a rigid skeleton network in the mode of point contact, so that a complex and connected pore-fracture system can be formed in the process of fracturing. Compared with terrigenous clastic quartz, maybe its fracturing effect is more effective.^26,27^ The authigenic siliceous minerals in the Longmaxi Formation shale have two sources. One is that siliceous organisms flourished and then died to form micritic quartz through dissolution, reprecipitation, or recrystallization. The other is that the volcanic materials were devitrified during the diagenesis to form aphanitic or micritic siliceous minerals.^24,29,30^
(3) Developed reservoir space. In the shale of “authigenic quartz–organic matter + carbonate + felsic + carbonate” lamina combination and “authigenic quartz–organic matter + carbonate + bioclastic” lamina combination, lamellar structures composed of organic matter and inorganic minerals through mutual superimposition are developed. The porosity measured in the experiment ranged from 5.4% to 6%, averaging 5.6%. The shale in the “authigenic quartz–organic matter + carbonate + felsic + carbonate” lamina combination has a high TOC and brittle mineral content. In addition, there are a large number of organic–inorganic pores, and three types of laminae are superimposed with each other to constitute an effectively connected pore system. The permeability of the shale sample is measured by means of the pulse decay method. It is indicated that compared with other lamina combinations, the felsic–carbonate lamina combination is significantly superior in shale permeability, with the maximum permeability up to 0.04 mD, and its average gas content is 7.4 m^3^/t. In the “authigenic quartz–organic matter + carbonate” lamina combination, shale pores are developed with a permeability of 0.03 mD and an average gas content of 6.1 m^3^/t.

In the environment with flourishing marine organisms, an “authigenic quartz–organic matter + carbonate + felsic–carbonate” lamina combination is formed. This combination has enriched organic matter; therefore, its hydrocarbon generation potential is quite great. Thanks to the vertical superimposition of multiple types of laminae, a connected system of abundant organic and inorganic pores is formed to provide favorable conditions for reservoir, seepage, and gas bearing property. What is more, the high brittle mineral content of authigenic quartz and carbonate provides very good fracability. Therefore, the interval with the developed “authigenic quartz–organic matter + carbonate + felsic–carbonate” lamina combination is the optimal “sweet spot layer” for shale gas development in the lower section of the Long 1_1_ submember. The shale reservoir with the “authigenic quartz–organic matter + carbonate” lamina combination takes second place in quality, and it is the secondary optimal “sweet spot layer” for the shale gas development in the lower section of the Long 1_1_ submember. The shale reservoir with the “carbonate + clay mineral” lamina combination is also good in quality, and it is the preferential “sweet spot layer” for shale gas development in the upper section.

## 7 Conclusions

(1) In the Zigong Block of southern Sichuan Basin, the Long 1_1_ submember exhibits six types of laminae, including authigenic quartz–organic lamina, carbonate lamina, felsic–carbonate lamina, terrigenous quartz lamina, clay mineral lamina, and pyrite lamina. These laminae form four distinct combinations based on their vertical distribution relationships, identified as “authigenic quartz–organic matter + carbonate + felsic–carbonate”, “authigenic quartz–organic matter + carbonate”, “carbonate + clay mineral”, and “terrigenous quartz + clay mineral”.
(2) The formation of felsic–carbonate lamina is under the direct effect of volcanics transportation and sedimentation. Felsic–carbonate lamina, authigenic quartz–organic lamina, and carbonate lamina interweave with each other to constitute “authigenic quartz–organic matter + carbonate + felsic–carbonate” lamina combination, which is mainly developed in the first sublayer of the Long 1_1_ submember. This lamina combination is regarded as the optimal lamina combination due to its high TOC and brittle mineral contents, developed pores, and high gas content. In addition, the corresponding shale interval is treated as the optimal “sweet spot layer” for the shale gas exploration and development in the lower section of the Long 1_1_ submember due to its favorable geological characteristics.
(3) The second sublayer of the Long 1_1_ submember features a lamina combination comprising “authigenic quartz–organic matter + carbonate”. While its TOC and brittle mineral contents, as well as pore development degree and gas content, are slightly lower compared with those of the “authigenic quartz–organic matter + carbonate + felsic–carbonate” lamina combination, it is regarded as a secondary optimal lamina combination. Consequently, its corresponding shale interval is identified as the secondary optimal “sweet spot” for the shale gas exploration and development in the lower section of the Long 1_1_ submember.
